# The COVID-19 pandemic: knowledge, attitudes and practices of coronavirus (COVID-19) among patients with type 2 diabetes

**DOI:** 10.1186/s41043-023-00349-7

**Published:** 2023-02-20

**Authors:** Hashem Mohamadian, Amrullah faraji, Ahmad Tahmasebi Ghorrabi, Kamel Ghobadi-Dashdebi, Arash Salahshouri

**Affiliations:** 1 Department of Health Education and Promotion, School of Health, Ahvaz Jundishapur University of Medical Sciences, Ahvaz, Iran; 2 Health Services Management, Ahvaz Jundishapur University of Medical Sciences, Ahvaz, Iran; 3 Health Care Management, School of Health Management and Information Sciences, Iran University of Medical Sciences, Tehran, Iran; 4 Department of Health Education and Promotion, School of Health, Isfahan University of Medical Sciences, Isfahan, Iran

**Keywords:** Knowledge, Attitude, Practice, Coronavirus, COVID-19, Type 2 diabetes

## Abstract

**Background:**

It is necessary to evaluate COVID-19 data on Knowledge, Attitudes and Practices (KAP) to confirm effective protective practice and to reduce risk in society. Hence, the study was carried out to determine KAP towards COVID-19 and the factors associated with knowledge and practices among patients with type 2 diabetes (T2D).

**Methods:**

In this cross-sectional (descriptive-analytical) study, 357 patients with diabetes in Izeh participated in the study. The sampling method used was convenience sampling method. Data collection tool was a researcher-made questionnaire of demographic information and KAP of patients with T2D in relation to the new coronavirus. The validity and reliability of the research tool was confirmed using the content validity and test–retest. Data analysis was done in Stata.14.2 and Smartpls 3.2.8 using descriptive and analytical statistical tests.

**Results:**

The mean score of participants' KAP towards Covid-19 was 74.22 (16.30), 72.88 (14.87), and 70.51 (19.70), respectively. The lowest and the highest score of the patients' knowledge was in the field of transmission (56.60 (20.96)) and care and prevention of the COVID-19 (88.58 (21.88)), respectively. Residence was the most important factor predicting the practice of diabetic patients with an explanatory coefficient ([SMD 1.08 (95% CI 0.85 to 1.30), *P* < 0.001] and *R*^2^ = 0.87%).

**Conclusion:**

Despite the good level of KAP of people towards the COVID-19 disease, there were answers showing poor knowledge, incorrect beliefs and attitudes, and insufficient practice regarding different aspects of the COVID-19 in some cases in our study. Residence was a strong predictor of type 2 diabetes mellitus (T2DM) patients’ practice in terms of protective behaviors against Covid-19. Hence, educational needs evaluation based on residence is recommended, especially in rural T2DM patients.

## Background

Severe acute respiratory syndrome of coronavirus (SARS-CoV-2) is the cause of the corona disease, first reported in the city of Wuhan, China, in December 2019, and since then it has spread to many countries in the world and the World Health Organization (WHO) declared it as a global pandemic on March 11, 2020 [1]. The disease spreads among humans through respiratory droplets of symptomatic and asymptomatic patients [2], with symptoms of fever, cough and fatigue [3–5]. Most of the patients recover without the need for special treatment, but the elderly and those with underlying diseases like cardiovascular diseases, diabetes, chronic respiratory disease and cancer are classified as high-risk people. They are more susceptible to septic shock, acute respiratory distress syndrome, electrolyte imbalance (metabolic acidosis) and coagulation disorders resulting in death ultimately [6–9]. According to previous studies, due to weak immune system [10], people with diabetes are at increased risk of severe disease, acute respiratory distress syndrome, and increased mortality in COVID-19 [11–14]. A study on 1099 patients with COVID-19 showed that 23.7% had high blood pressure and 16.2% had diabetes out of 173 patients with severe disease [15]. Another study on 52 patients with severe COVID-19 showed that 7 (22%) out of 32 patients who died had diabetes [6]. Additionally, the results of a study on 7,337 patients with SARS-CoV-2 in China showed that T2D significantly increases the risk of death in hospitalized patients with COVID-19 [16]. During the covid-19 pandemic, the relationship between diabetes and the contracting covid-19 has been closely examined. In these surveys, it was confirmed that because patients with type 2 diabetes are more at risk of contracting pneumonia than others, the possibility of contracting Covid-19 and especially severe forms of the disease is higher [17–19]. An interactive relationship between diabetes and COVID-19 was also proven. On the one hand, diabetes is associated with an increased risk of severe SARS-CoV-2 infection, and on the other hand, both severe metabolic complications of new diabetes and previous diabetes have been observed in patients with COVID-19 [18, 20, 21].

Besides therapeutic interventions and vaccination, non-clinical interventions such as limiting unnecessary travel, observing social distance, wearing masks, and frequent and proper hand washing have been suggested by the WHO to control the spread of COVID-19 [22, 23]. However, according to the “KAP theory”, the adherence of patients with diabetes to these control measures will be largely affected by their knowledge, attitude and practice (KAP) towards COVID-19 [24]. “KAP theory” is a theory of health-related behavior change, where the change in human behavior is affected by three consecutive processes: acquiring knowledge, creating attitude and adopting behavior (or practice) [25]. Moreover, perceived knowledge and attitude are important predictors of adherence to health behaviors [26]. It must be noted that a misunderstanding of an emerging disease combined with insufficient specialist knowledge can lead to fear and chaos and exacerbate the epidemic. As past experiences reveal, misconceptions and excessive fear in people have resulted in resistance to compliance with public health control measures and contributed to the rapid spread of diseases [2, 27, 28]. Also, several studies have confirmed the relationship between the levels of knowledge and attitude in patients with preventive behaviors [29–31]. Therefore, the KAP levels of people with diabetes were expected to be an important determinant in their fight against COVID-19. On the other hand, it was thought that the perceptions and behaviors of patients with diabetes were affected by the COVID-19 epidemic, which needed to be investigated. Thus, understanding the knowledge, attitude and practice of high-risk groups, especially patients with diabetes, seems to be a necessary thing that can help predict the results of planned behavior in COVID-19 patients. Hence, the study was performed to determine KAP in relation to COVID-19 and factors associated with knowledge and practice among patients with T2D. The results could inform trainings, policies and other effective and important intervention strategies for better and timely containment of COVID-19 and put patients with T2D in the priority plans of public health officials, doctors and the country media.

## Materials and methods

### Study design

This was a cross-sectional (descriptive-analytical) study that conducted between October 2021 and January 2022 for examining KAP of the new COVID-19 in patients with T2D in Izeh, Iran.

### Study setting and sites

Izeh is one of the cities of Khuzestan located in the south of Iran. The study was carried out in 24 comprehensive health service centers of Izeh.

### Participants and sample size

The population examined was all patients with T2D in Izeh. According to G-power software using an exploratory a priori approach (a 2-tailed test, *α* = 0.05, *β* = 0.2, allocation ratio of *N*2/*N*1 = 0.65, and the effect size (Cohen's d) of 0.3), the sample size for this study was 368. Finally, 357 patients participated in the study and answered the questions of the questionnaire. The sample selection method was multi-stage based on multiple urban and rural clusters. Thus, first, 4 urban and 4 rural centers were considered as 8 clusters. In the next step, the researchers visited comprehensive health service centers and obtained information from the registers of patients with T2D. They determined the number of patients needed according to the proportion of patients in each center from the total sample size. Finally, the samples of each center were selected from the people who went to the centers to receive services through convenient sampling. Overall, 226 people were selected from 4 urban and 131 people from 4 rural centers.

### Data collection

A researcher-made structured questionnaire was used for data collection. Trained interviewers were used to complete the questionnaires. For this purpose, a coordination meeting was held with the questioners and after explaining the goals of the project, they were taught how to complete the questionnaires. The questionnaires were completed as self-expressed by the routine clients of the centers. To increase the accuracy of the individuals in completing the questionnaires, they were given the necessary explanations about the questions before completing the information. In specific cases, the questionnaires were completed in the form of a face-to-face interview (following health protocols) if people were not able to complete the information. The inclusion criteria were a history ofT2D at least 3 months before the outbreak of the epidemic and a complete answer to the questions in the questionnaire. Exclusion criteria were lack of satisfaction to participate in the study and physical and mental problems.

### Measurement tools

The questionnaire had four sections. The first part has 12 questions in the field of demographic characteristics such as age, gender, occupation, education, residence, marital status, and time of diagnosis.

The second part has 31 questions to measure the knowledge of patients with T2D in relation to COVID-19. Knowledge questions were divided into 4 sub-components (Knowledge about the Nature of the coronavirus and the Disease (KNCD) (6 questions), Knowledge about the transmission of the coronavirus (KT) (4 questions), Knowledge about the Care and Prevention of the coronavirus (KCP) (10 questions), Knowledge about coronavirus diagnosis and treatment (KDT) (11 questions). The score range of each question is between 0 and 2 (correct = 2, don't know = 1 and wrong answer = 0).

The third part has 8 questions associated with patients' attitude towards COVID-19. The questions in this section are divided into 4 sub-components (Attitude towards Contracting the disease (AC); Attitude towards Severity and Mortality of the Disease (ASMD); Attitudes towards Disease Prevention (ADP); Attitudes towards Disease Treatment (ADT)). The questions in this section were designed based on a 3-point Likert scale with a score range of 0 to 2 for each question (disagree = 0, have no opinion = 1, and agree = 2).

The last part has questions about patients' Practice regarding COVID-19 (PC) (11 questions). The questions in this section were based on a 3-point Likert scale with a score range of 0 to 2 for each question (not at all = 0, somewhat = 1, and very much = 2).

### Validity and reliability of the questionnaire

The basic questions of the questionnaire have been prepared based on the instructions of the Ministry of Health, Treatment and Medical Education of Iran in the field of prevention and control of COVID-19 [15]; as well as the texts of similar papers [3, 6, 24, 32–36]. The validity of the tool was examined in two ways: face validity and content validity. Seven experts in the field of health education and health promotion and infectious disease experts were interviewed in a group discussion and face to face to examine the face validity of the tool, and their opinions about the level of difficulty, the degree of inadequacy, the ambiguity of expressions or the existence of insufficiency in meanings of the words of the questionnaire were taken into account.

In qualitative content validity, 9 experts were asked with specializations in health education and health promotion (5 people), infectious diseases specialist (2 people) and internal medicine specialist (2 people). After a detailed study of the tool, they presented their corrective views about each item in detail and in writing. Necessary changes were made in the tool after collecting comments.

Content validity ratio (CVR) and the content validity index (CVI) were used in a quantitative method to determine the content validity. Based on the results obtained from the opinions of 9 experts, the overall CVR value of the questionnaire was 0.90–0.88 for knowledge, 0.90 for attitude, and 0.92 for practice. Moreover, CVI value for all sections of the questionnaire was calculated by averaging the questions of each section, where knowledge was 0.93, attitude 0.91, and practice 0.89. The overall CVI value of the questionnaire was 0.91.

### Reliability of the questionnaire

Retest method was used to determine the reliability. Therefore, the validated version of the questionnaire was given to 20 patients with T2D. One week later, they were asked to complete the questionnaires again. The reliability rate for the entire questionnaire was 0.91 (0.87 for knowledge, 0.92 for attitude and 0.94 for practice), which was within the acceptable range. Ultimately, the tool was prepared as a questionnaire with 50 items in three sections.

### Statistical analysis

Univariate and multivariate data analysis was mainly used in the study. Qualitative variables were analyzed using frequency and percentage, and quantitative variables were analyzed using mean and standard deviation or, if necessary, median and range using Stata.14.2. Shapiro–Wilk Test was used to examine the normality of the data. Independent t-test was used with the help of effect size index (SMD) for knowledge, attitude and practice and their dimensions for intergroup comparison. Then multiple linear regression analysis was used to confirm the relationships between the variables. Moreover, Smartpls 3.2.8 was used to draw relationships between knowledge about COVID-19 in patients with T2D and its sub-components according to place of residence. All the tests were statistically measured at the error level of 5%.

## Results

### Demographic characteristics of study participants

Furthermore, 357 patients participated in the study. The mean age of the study participants was 57.8 (12.0) years, 64.1% (229 people) of the participants were female and 89.4% (319 people) were married. Other demographic characteristics of the patients are given in Table 1.

**Table 1:** Demographic characteristics of patients with T2D (n = 357)

	Variables	Frequency (%)
Sex	Female	229 (64.1)
	Male	128 (35.9)
Residence	Urban	226 (63.3)
	Rural	131 (36.7)
Marital	Married	319 (89.4)
	Others	38 (10.6)
Educational	Illiteracy	241 (67.5)
	Literacy	116 (32.4)
Job	Housewife	213 (59.7)
	Others	144 (40.3)
Number of Children	<=3 Child	82 (23)
	>3 Child	275 (77)
Duration of Diagnosis	<=5 Years	42 (56)
	>5 Years	157 (44)
Complications of Diabetes	Kidney Problems	63 (17.6)
	Nervous Problems	71 (19.9)
	Blurred Vision	38 (10.6)
	Vision Problems	21 (5.9)
	Stroke	4 (1.1)
	High Blood Pressure	172 (48.2)
	High Blood Fats	138 (38.7)
History in First-Degree Relatives	Yes	203 (56.9)
	No	154 (43.1)
History of Infection	Yes	160 (44.8)
	No	197 (55.2)

Based on Table 2, the mean and standard deviation of the knowledge score of the participants regarding the COVID-19 was 74.22 (16.30). The lowest mean knowledge score was (56.60 (20.96)) regarding coronavirus transmission and the highest mean knowledge score (88.58 (21.88)) was in care and prevention. It is noteworthy that the mean knowledge score of the participants in all components had a significant difference based on residence, which were in the range of moderate and strong relationship in terms of the effect size index. The mean knowledge score of the participants according to the type of drug consumed except knowledge about the transmission of the coronavirus showed a significant difference with the mean effect size. Moreover, the mean knowledge score of the participants according to their marital status showed a significant difference with the mean effect size apart from the component of care, prevention and transmission of the virus. Although there was a significant difference in the mean knowledge score of the participants according to the level of education in all components, there is a weak relationship with regard to the care and prevention, diagnosis and treatment of the coronavirus in terms of the effect size index except for the knowledge components and there is a moderate relationship in the other components. Additionally, the mean knowledge score of the participants based on the history of diabetes in first-degree relatives was significantly different in all components, but in terms of the effect size index, in the component of knowledge about care and prevention, it showed a weak relationship and in the component of knowledge about the transmission of the coronavirus, it showed a moderate relationship. Although the mean knowledge score of the participants in terms of age showed a significant difference in all components, they were in the range of weak relationship for the effect size index. It has to be noted that the mean knowledge score of the participants in terms of gender was insignificantly different in all components, and they were within the noticeable range in terms of the effect size index. The mean knowledge score of the participants in terms of the duration of diagnosis of diabetes was insignificantly different only in the component of knowledge about the diagnosis and treatment of the coronavirus (although it was in the medium range in terms of the effect size index), there was a significant difference with the effect size being insignificant and weak in other components.

**Table 2:** Knowledge of patients with T2D regarding COVID-19 (n = 357)

	Variable	KNCVD Mean (SD)	KT Mean (SD)	KCP Mean (SD)	KDT Mean (SD)	GK Mean (SD)
				Mean (SD)	Mean (SD)	Mean (SD)
Sex	Man	81.15 (25.48)	55.66 (23.46)	86.19 (25.09)	71.73 (19.40)	73.68 (18.77)
	Women	81.58 (22.71	57.12 (19.45)	89.92 (19.80)	69.46 (18.06)	74.52 (14.78)
	MD [95% CI]	− 0.43 [− 5.59 to 4.72]^ns^	− 1.46 [− 6.01to 3.10]^ns^	− 3.73 [− 8.47 to 1.00]^ns^	2.27 [− 1.76 to 0.30]^ns^	− 0.83 [− 4.38 to 2.70]^ns^
	Cohen’s d [SMD]	− 0.19 [−0.23 to 0.20]^ns^	− 0.07 [− 0.29to .15]^ns^	− 0.17 [− 0.39 to .05]^ns^	0.12 [− 0.1 to 0.33]^ns^	− 0.05 [− 0.26 to 0.16]^ns^
Age	<58 years	56.35 (20.03)	58.77 (21.96)	91.97 (17.13)	73.67 (16.54)	77.69 (13.98)
	>58 years	76.54 (26.02)	54.43 (19.74)	85.22 (25.37)	66.90 (19.85)	70.77 (17.69)
	MD [95% CI]	9.82 [4.98 to 14.65]^**^	4.34 [− 0.002 to 8.69]^*^	6.74 [2.23 to 11.25]^**^	6.77 [2.97to10.58]^**^	6.91 [3.60to 10.24]^**^
	Cohen’s d [SMD]	0.42 [0.21 to 0.63]^**^	0.20 [− 0001 to 0.42]^*^	0.31 [0.10 to 0.51]^**^	0.37 [0.16 to 0.57]^**^	0.43 [0.22 to 0.64]^**^
Residence	Urban	87.76 (16.41)	6.48 (21.00)	94.17 (12.03)	77.36 (14.68)	79.94 (10.53)
	Rural	70.51 (29.73)	49.90 (19.20)	78.95 (30.22)	58.05 (18.24)	64.35 (19.52)
	MD [95% CI]	17.24 [12.44to22.04^**^	10.57 [6.17to14.98]^**^	15.21 [10.76to19.68]^**^	19.3 [15.84to22.78]^**^	15.59 [12.46to 18.72]^**^
	Cohen’s d [SMD]	0.77 [0.55 to 0.99]^**^	0.51 [0.30to 0.73]^**^	0.74 [0.50 to 0.96]^**^	1.20 [0.97 to 1.43]^**^	1.08 [0.85 to 1.30]^**^
Diabetes in relatives	Yes	87.43 (17.92)	60.69 (21.82)	92.58 (16.46)	74.28 (16.59)	78.81 (12.82)
	No	73.51 (27.81)	50.85 (18.30)	83.31 (26.60)	64.99 (19.71)	68.16 (18.35)
	MD [95% CI]	13.93 [9.15to18.70]^**^	10.10 [5.82to14.40]^**^	9.27 [4.77 to 13.77]^**^	92.9 [5.5 to 13.07]^**^	10.46 [7.40 to 3.90]^**^
	Cohen’s d [SMD]	0.61 [0.39 to 0.82]^**^	0.49 [0/28 to 0.71]^**^	0.43 [0.22 to 0.64]^**^	0.5 [0.30 to 0.72]^**^	0.69 [0.47 to 0.90]^**^
Educational status	Illiteracy	76.66 (25.59)	53.03 (20.24)	85.73 (24.50)	67.40 (18.77)	70.70 (17.02)
	Literacy	91.34 (15.08)	64.01 (20.58)	94.50 (13.34)	76.25 (16.67)	81.53 (11.77)
	MD [95% CI]	− 14.68 [− 19.73 to − .42]^**^	− 10.97 [15.5 to − 0.45]^**^	− 8.87 [− 13.55 to 3.98]^**^	− 8.85 [− 12.88 to − 0.82]^**^	− 10.82 [− 14.26 to − 7.37]^**^
	Cohen’s d [SMD]	− 0.65 [− 0.87to − 0.42]^**^	− 0.54 [− 0.76 to − 0.31]^**^	− 0.41 [− 0.63 to − 0.18]^**^	− 0.49 [− 0.71 to − 0.26]^**^	− 0.70 [− 0.92 to − 0.47]^**^
Marital status	married	82.85 (22.28)	56.97 (21.04)	89.06 (20.48)	71.60 (18.10)	75.12 (15.44)
	other	69.52 (31.29)	53.45 (20.22)	84.60 (31.40)	59.15 (18.84)	66.68 (21.09)
	MD [95% CI]	13.33 [5.44 to 21.22]^**^	3.52 [− 3.55 to 10.60]^ns^	4.45 [− 2.92 to 11.83]^ns^	12.45 [6.3 to 18.56]^**^	8.44 [2.10 to 13.88]^**^
	Cohen’s d [SMD]	0.57 [0.23 to 0.90]^**^	0.16 [− 0.16 to 0.50]^ns^	0.20 [− 0.13 to 0.54]^ns^	0.68 [0.34 to 1.02]^**^	0.52 [0.18 to 0.86]^**^
Duration of diagnosis	<5 years	83.35 (23.32)	59.96 (22.02)	91.21 (20.22)	70.71 (17.88)	76.31 (15.51)
	>5 years	78.98 (24.06)	52.30 (18.72)	85.24 (23.48)	69.72 (19.42)	71.56 (16.95)
	MD [95% CI]	4.37 [− 0.58 to 9.33]^*^	7.65 [3.33 to 11.99]^**^	5.97 [1.41 to 10.52]^**^	0.99 [− 2.9 to 40.9]^ns^	4.75 [− 1.36 to 8.13]^**^
	Cohen’s d [SMD]	0.18 [− 0.02 to 0.39]^*^	0.37 [0.16 to 0.58]^**^	0.27 [0.06 to 0.48]^**^	0.53 [− 0.16 to 0.26]^ns^	0.29 [0.84 to 0.50]^**^
Medication	metformin	77.95 (26.48)	56.31 (21.36)	85 (25.87)	67.18 (18.68)	71.61 (19.14)
	other	86.39 (18.03)	57.01 (20.45)	93.71 (12.86)	74.69 (17.51)	77.95 (9.10)
	MD [95% CI]	− 8.43 [− 13.38 to − 0.49]^**^	− 0.70 [− 0.14 to 3.73]^ns^	− 8.7 [− 13.25 to − 4.16]^**^	− 7.50 [− 11.35 to − 3.65]^**^	− 6.34 [− 9.73 to − 2.94]^**^
	Cohen's d [SMD]	− 0.36 [− 0.57 to − 0.14]^**^	− 0.03 *[− 0.24 to 0.17]^ns^	− 0.40 [− 0.61 to − 0.19]^**^	− 0.41 [− 0.62 to − 0.20]^**^	− 0.39 [− 0.61 to − 0.18]^**^

*(*p* value < 0.5); ** (*p* value < 0.01)

**Fig. 1:**
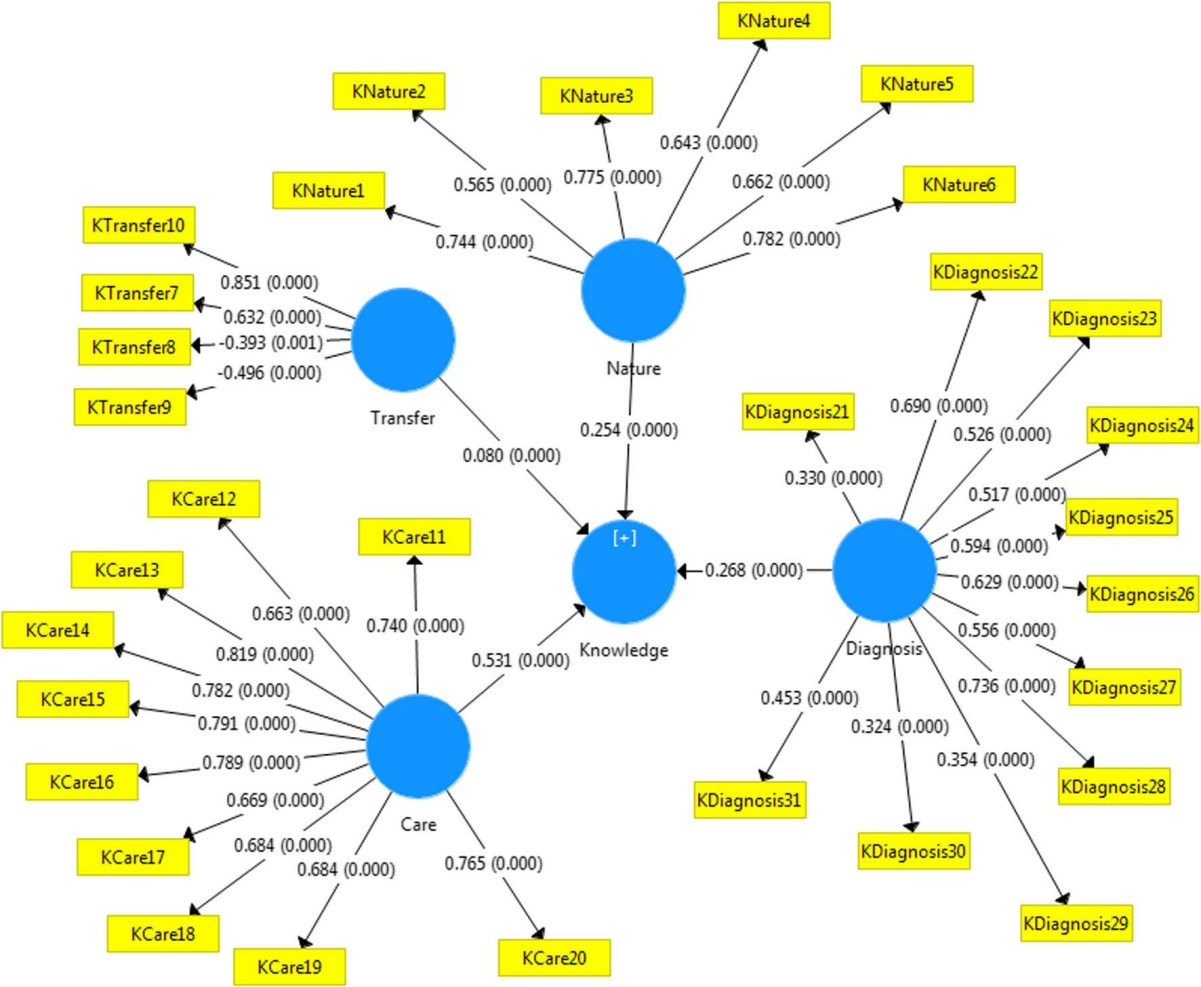
Determining the relationship between sub-components of knowledge according to residence in patients with type 2 regarding COVID-19

As residence was an effective determinant in predicting the knowledge of the participants, this diagram was drawn using Smart PLS3.2.8 for a better understanding of the readers (Fig. 1).

As Table 3 shows, the mean and standard deviation of the overall attitude score of the patients towards the disease was 72.88 (14.87). The analysis of the results in the sub-components revealed that the lowest mean attitude score was for attitude towards the treatment of the COVID-19 (56.60 (20.96)) and the highest for attitude towards the disease (88.58 (21.88)).

**Table 3:** Attitude of patients with T2D towards COVID-19 (n = 357)

Variable		AC Mean (SD)	ASMD Mean (SD)	ADP Mean (SD)	ADT Mean (SD)	GA Mean (SD)
Sex	Man	76.17 (32.58)	90.04 (19.92)	69.92 (22.04)	54.30 (28.77)	71.48 (16.37)
	Women	82.09 (31.87)	93.67 (17.15)	72.49 (23.69)	54.37 (28.82)	73.66 (13.94)
MD [95% CI]		− 9.92 [− 12.89 to 1.04]^*^	− 3.63 [− 7.58 to 0.31]^*^	− 2.57 [− 7.59 to 2.45]^ns^	− 0.07 [− 6.32 to 6.18]^ns^	− 2.18 [− 5.4 to 1.04]^ns^
Cohen's d [SMD]		− 0.18 [− 0.40 to 0.32]^*^	− 0.20 [− 0.41 to 0.01]^*^	− 0.11 [− 0.33 to 0.11]^ns^	− 0.002 [− 0.22 to 0.21]^ns^	− 0.14 [− 0.36 to 0.07]^ns^
Age	<58 years	80.90 (32.62)	93.68 (16.55)	65.17 (21.30)	47.19 (27.34)	76.97 (15.02)
	>58 years	79.05 (30.80)	91.06 (19.75)	77.93 (23.14)	61.45 (28.44)	68.82 (13.59)
MD [95% CI]		1.85 [− 4.86 to 8.56]^ns^	2.62 [− 1.18 to 6.41]^ns^	− 12.76 [− 17.4 to − 8.13]^**^	− 14.26 [− 20.06 to − 8.45]^**^	8.15 [5.16 to 11.13]^**^
Cohen's d [SMD]		0.05 [− 0.15 to 0.26]^ns^	0.14 [− 0.061 to 0.35]^ns^	− 0.57 [− 0.78 to − 0.36]^**^	0.51 [− 0.72 to − 0.29]^**^	0.56 [035 to 0.78]^**^
Residence	Urban	87.38 (28.01)	94.24 (14.72)	67.69 (22.53)	57.08 (29.43)	75.80 (14.28)
	Rural	67.17 (34.95)	89.12 (22.81)	78.24 (22.65)	49.62 (27.03)	67.84 (14.57)
MD [95% CI]		20.21 [13.58 to 26.85]^**^	5.12 [1.22 to 9.04]^**^	− 10.54 [− 15.42 to − 5.67]^**^	7.46 [1.28 to 13.63]^**^	79.96 [4.85 to 11.06]^**^
Cohen's d [SMD]		0.66 [0.44 to 0.88]^**^	0.28 [0.07 to 0.50] ^**^	− 0.46 [− 0.68 to − 0.24]^**^	0.26 [0.04 to 0.47]^**^	0.55 [0.33 to 0.77]^**^
Illiteracy	Educational status	76.97 (34.13)	91.28 (19.68)	74.69 (32.21)	56.74 (29.29)	70.33 (15.04)
Literacy		86.21 (26.85)	94.61 (14.74)	65.09 (21.59)	49.35 (27.08)	78.18 (13.07)
MD [95% CI]		− 9.24 [− 16.34 to − 2.13]^**^	− 3.32 [− 7.37 to 0.72]^**^	9.6 [4.56 to 14.64]^**^	7.39 [1.03 to 13.74]^**^	− 7.84 [− 11.05 to − 4.64]^**^
Cohen's d [SMD]		− 0.28 [− 0.51 to 0.− 0.06]^**^	− 0.18 [− 0.40 to 0.04]^**^	0.42 [0.20 to 0.65]^**^	0.26 [0.03 to 0.48]^**^	− 0.54 [− 0.77 to − 0.31]^**^
Marital status	Married	81.19 (31.83)	93.26 (17.10)	71.32 (23.03)	55.09( 28.52)	73.35 (14.75)
	Other	69.74 (33.97)	84.86 (25.03)	73.68 (23.93)	48.03 (30.41)	68.91 (15.48)
MD [95% CI]		11.45 [0.63 to 22.27]^**^	8.39 [2.28 to 14.50]^**^	− 2.37 [− 10.17 to 5.44]^ns^	7.08 [− 2.62 to 16.76]^ns^	4.43 [− 0.56 to 9.44]^**^
Cohen's d [SMD]		0.36 [0.01 to 0.69]^**^	0.46 [0.12 to 0.80]^**^	− 0.10 [− 0.44 to 0.23]^ns^	0.25 [− 0.09 to 0.58^ns^	0.29 [− 0.04 to 0.64]^**^
Number of	>3 child	78.91 (32.68)	92 (18.73)	73.8 (22.93)	54.54 (28.04)	72.23 (14.52)
	<3 child	83.54 (30.48)	93.60 (16.58)	66.16 (23.03)	53.66 (31.21)	75.08 (15.88)
MD [95% CI]		− 4.62 [− 12.59 to 3.34]^ns^	− 1.60 [− 6.12 to 2.92]^ns^	7.02 [1.34 to 12.70]^**^	0.89 [− 6.24 to 8.01]^ns^	− 2.84 [− 6.52 to 0.82]^ns^
Cohen's d [SMD]		− 0.14 [− 0.39 to 0.11]^ns^	− 0.09 [− 0.33 to 0.16]^ns^	0.30 [0.06 to 0.55]^**^	0.03 [− 0.21 to 0.28]^ns^	− 0.19 [− 0.43 to 0.05]^ns^
Medication	Metformin	78.81 (34.46)	90.83 (20.37)	66.55 (21.57)	51.67 (25.24)	73.42 (15.61)
	Other	81.63 (28.72)	94.55 (14.49)	78.74 (23.11)	58.16 (32.86)	72.81 (13.85)
	MD [95% CI]	− 2.82 [− 9.64 to 3.99]^ns^	− 3.72 [− 7.57 to 0.12]^**^	− 12.19 [16.92 to − 7.46]^**^	− 6.5 [− 12.55to− 0.44]^**^	1.31 [− 1.83 to 4.46]^ns^
	Cohen's d [SMD]	− 0.08 [− 0.29 to 0.12]^ns^	− 0.20 [− 0.41 to 0.01]^**^	− 0.54 [− 0.76 to − 0.33]^**^	− 0.23 [− 0.44 to − 0.01]^**^	0.08 [− 0.12 to 0.30]^ns^

*(*p *value < 0.5); ** (*p *value < 0.01)

There were no significant differences in the mean score of the participants' attitude according to gender in all components, and they were in the negligible range in terms of the effect size index.

The mean score of the attitude of the participants according to age in the components of the attitude towards care and prevention and the attitude towards the treatment had a significant difference and in terms of the effect size index, it was in the range of the mean relationship. Moreover, the mean score of the participants' attitude in all components showed a significant difference according to residence, and they were in the range of weak and moderate relationship in terms of the effect size index.

The mean attitude score of the participants according to the educational status in all components (except the component of attitude towards the severity and mortality of the disease) showed a significant difference with the effect size of the weak relationship. Moreover, the mean attitude score of the participants based on marital status in all components (except care and prevention and attitude towards disease treatment) showed a significant difference with the effect size of the weak relationship.

The mean score of the participants' attitude according to the number of children showed a significant difference only in the attitude component of the ratio of care and prevention of corona disease, and in terms of the effect size index, it was in the weak range.

The mean score of the participants based on the type of drug used in all components except the attitude towards contracting the coronavirus and also the attitude towards the severity and mortality of the disease showed a significant difference with the effect size of the weak and moderate relationship.

As Table 4 shows, the mean and standard deviation of the patients' practice score was 19.70 (1970.51). Although this situation had no significant differences in terms of gender, it was in the range of a strong relationship in terms of the effect size index.

**Table 4:** The practice of patients with T2D compared to COVID-91 disease

Practice		Mean (SD)	Md [95% CI]	Cohen's D [SMD]	*T* value	*P* value
Sex	Man	69.39 (18.83)	− 1.74 [− 6.02 to 2.53]	− 0.88 [− 0.30 to 0.13]	0.8	0.44
	Women	71.14 (20.19)				
Residence	Urban	77.83 (18.80)	19.93 [16.21 to 23.66]	1.15 [0.92 to 1.39]	10.54	0
	Rural	57.89 (14.07)				
Diabetes in First-Degree Relatives	Yes	73 (19.44)	5.75 [1.65 to 9.86]	0.29 [0.08 to 0.51]	2.75	0.006
	No	67.24 (19.64)				
Educational	Illiteracy	68.37 (19.54)	− 6.60 [− 10.92 to − 2.26]	− 0.34 [− 0.56 to − 0.11]	− 3	0.03
	Literacy	74.96 (19.38)				
Marital	Married	71.33 (19.44)	7.66 [1.05 to 14.27]	0.39 [0.05 to 0.73]	2.28	0.02
	Other	63.66 (20.86)				
Number of Children	> 3 Child	68.78 (19.81)	− 7.54 [− 12.36 to − 2.71]	− 0.39 [− 0.63 to − 0.14]	− 3.07	0.002
	< 3 Child	76.32 (18.29)				
Medication	Metformin	66.53 (18.52)	− 9.67 [− 13.72 to − 5.62]	− 0.50 [− 0.72 to − 0.29]	− 4.69	0
	Other	76.20 (20.02)				

The mean practice score of the participants showed a significant difference based on residence, and it showed a very strong relationship in terms of the effect size index. Moreover, the mean practice score of the participants based on the history of diabetes in the first-degree family, educational status, marital status, number of children, and the type of drug used showed a significant difference, but showed a weak relationship in terms of the effect size index.

Multiple linear regression analysis of the relationship between age, gender, residence, attitude towards infection, attitude towards treatment, general attitude of patients, knowledge about disease diagnosis and treatment, knowledge about care and prevention, education status, type of drug used, with patients' practice in follow-up behaviors of COVID-19 showed as follows:

*Y* = 68.34 + 0.22 Age + 3.79 Sex − 15.52 residence + 0.09 Att_1_ − 0.11 Att_4_ − 0.35 Att + 0.41 Kdt − 0.18 KCP + 6.52 Educational status + 5.73 Medication

In multiple regression analysis, to predict the practice of patients with T2D in terms of preventive behaviors against COVID-19 based on the variables examined, the results showed that the most important factors predicting the practice score of the patients were, respectively, residence (*P* < 0.001) and knowledge about the diagnosis and treatment of the virus (*P* < 0.001) (Table 5).

**Table 5:** The Crude and standardized coefficients of the regression line of the practice of patients with T2D in the field of preventive behaviors against COVID-19 according to the variables examined

Variable	Crude coefficients	Standardized coefficients	*P* value
Constant	68.34		
Age	0.22	0.13	0.015
Sex	3.79	0.2	0.035
Education status	6.52	0.33	0.004
Residence	− 15.52	0.78	< 0.001
Medication	5.73	0.3	0.002
Attitude towards Contracting the disease	0.09	0.14	0.018
Attitudes towards Disease Treatment	−0.11	0.16	0.031
General attitude	−0.35	0.26	0.004
Knowledge about coronavirus diagnosis and treatment	0.41	0.38	< 0.001
Knowledge about the Care and Prevention	− 0.18	0.2	0.001

Examining the relationship between the practice of patients with knowledge (and its subscales) and attitude towards the disease in Table 6 indicated a significant positive relationship between the practice of the participants and their knowledge about the diagnosis and treatment of the disease (*r* = 0.429, *p* < 0.001). Thus, as the knowledge of the participants increased, their performance improved (although the type of relationship is weak); nonetheless, there were no significant relationships between the performance of the patients and the attitude towards the disease. Nevertheless, there was a positive and significant relationship between the attitude score of patients with all aspects of knowledge concerning COVID-19.

**Table 6:** Examining the relationship between knowledge (and its subscales) and attitude with the practice of patients with T2D towards the COVID-19 disease

		KNCVD	KT	KCP	KDT	GA	PC
KNCVD	r	1					
	95% Conf. Interval	–					
KT	r	0.312 **	1				
	95% Conf. Interval	0.22 to 0.40	–				
KCP	r	0.672 **	0.288 **	1			
	95% Conf. Interval	0.61 to 0.73	0.20 to 0.38	–			
KDT	r	0.566 **	0.282 **	0.539 **	1		
	95% Conf. Interval	0.50 to 0.63	0.18 to 0.38	0.47 to 0.61	–		
GA	r	0.494 **	0.392 **	0.543 **	0.298 **	1	
	95% Conf. Interval	0.41 to 0.57	0.30 to 0.48	0.47 to 0.61	0.20 to 0.39	–	
PC	r	0.244 **	0.104 *	0.09	0.429 **	0.004	1
	95% Conf. Interval	14 to 0.34	0.001 to 0.21	− 0.01 to 0.20	0.34 to 0.51	− 0.10 to 0.11	–

*p < 0.05, **p < 0.01

## Discussion

Nowadays, the prevalence of diabetes has brought the significance of this disease as a public health problem to attention [37]. Studies indicate that having underlying diseases like diabetes shows a poor prognosis in patients with COVID-19 [24, 38, 39], so that in COVID-19 patients, having diabetes mellitus is associated with increased complications and mortality [12, 14]. Under such conditions, people with diabetes should be cautious and take necessary precautions to prevent COVID-19. Thus, communities must follow accepted infection control practices such as frequent hand washing with soap, use of alcohol to disinfect hands, social distancing, knowledge of disease symptoms, vaccination, and use of masks to minimize the risk of transmission [39]. It is necessary to evaluate the data of COVID-19 on KAP to confirm effective protective practice and reduce risk in the community. This data is critical in providing the approaches needed to contain the spread of the virus. As far as we know, this study is among the limited studies that evaluate KAP of T2DM patients toward COVID-19.

According to the findings, the mean score of KAP was higher than 70 that despite being considered a good score is lower than the study by Ebrahimi et al. [40] and Kakemam et al. [41]. However, in Swain et al., about 78% of T2DM patients had mean knowledge and 10% of them had lower than mean knowledge [42]. This shows the low level of knowledge of T2DM patients. Given the time of our study, which was almost 2 years after the outbreak of COVID-19 in Iran, and the large volume of educational programs implemented via various channels (such as health service personnel, radio and television, and educational campaigns), prediction of the desired knowledge of society from disease was possible to some extent. In this regard, Gao et al. (2020) [43] indicated that most people had good knowledge about COVID-19. In Zhong et al., the awareness of COVID-19 in Chinese people was much higher than in our study (90%). However, unlike Zhong et al., 67.5% of our sample was illiterate [44]. Nonetheless, the high level of knowledge about the disease can be because of the characteristics of our sample. This is because with the warning of various media about the poor prognosis of patients with diabetes in case of COVID-19 can motivate this segment of the society to search for information on COVID-19.

According to data analysis, the lowest mean was associated with “Knowledge of the ways of transmission of COVID-19”. Thus, 21% thought that corona is transmitted by insect bites and 30.3% thought that corona is transmitted to a person with diabetes through an insulin injection needle. Not in line with the results of this study, another study in Iran on the industrial workers of Saveh showed that 88.9% of the samples were aware of the ways of transmission of COVID-19 [45]. One year before our study, the results of a study in one of Iran's cities showed that the lowest scores were associated with the lack of knowledge about the increased risk of contracting unprotected direct contact with pets and surfaces in contact with animals [46]. All these indicate a need for serious training in connection with the ways of disease transmission. The possible reason could be because of the confusion resulting from the conflicting content of the numerous educational resources made available to the public in connection with the COVID-19 and its transmission paths.

The study indicated that the highest score was associated with “knowledge about the care and prevention of COVID-19”. This could be because of the increased sensitivity of people to take preventive measures to protect their health. Although the patients' knowledge about care and prevention was somewhat favorable, a more detailed analysis of the results shows that only 5% of the samples answered the question “70% alcohol can destroy the corona virus” as “no”. Furthermore, 5% did not consider wearing masks 4.2% washing hands, 6.4% observing social distance, and 4.2% vaccination effective in the transmission and prevention of the virus, and 8.1% of them did not consider underlying diseases such as diabetes to be susceptible to corona. Although the poor knowledge of some patients with T2D about “ways to care for and prevent the coronavirus” seems a bit strange after a long time has passed since the corona epidemic, this can affect their practice of environmental measures to prevent the COVID-19 and cause more incidence of covid-19 in these patients. Our results in the practice section and their percentage of COVID-19 (44.8) confirm this.

Although the general analysis of the results in Table 2 shows “knowledge about the diagnosis and treatment of the coronavirus” as favorable, a more detailed analysis shows that 40.9% of patients consider the role of “smoking and drug use in eliminating the coronavirus” effective. Respectively, 10.9% and 7% of them were unaware of the role of Chloroquine Phosphate and Remdesivir in the treatment of the disease. Moreover, 55.5% of them still did not know that CT scan and blood test are ways to definitively diagnose the disease and a significant percentage of them were unaware of the role of vaccines in preventing the development of severe and fatal disease. Lack of knowledge in different fields of the disease can affect the attitude of patients in the field of preventive behaviors against corona. In confirmation of this statement, it is reminded that the results of the study in Table 6 confirmed a positive and significant relationship between the knowledge score and the attitude of patients in the field of preventive behaviors against corona.

One of the critical elements found in the current study was the role of residence on the acquired knowledge of patients so that patients living in the city and with higher education had better knowledge than others. These are similar to the findings of Erfani et al. [47] and Zhong et al. [44]. Moreover, Kasemy et al. indicated that a low knowledge score is associated with rural residence and low education [48]. Hosseinkhani in Iran indicated the relationship between people's level of knowledge about the COVID-19 and their level of education [36]. One can state that people living in urban society are usually literate and have the skills to use virtual space to search for the information they need, and on the other hand, they have easier access to information campaigns through social, digital or print media. Hence, compared to people living in rural and remote areas, they most likely have a higher level of knowledge about diseases. Thus, it is logical that awareness programs about COVID-19 should be directed towards specific demographic groups such as people with lower educational status and residents of rural areas.

Although the majority of our sample were females, no gender difference was seen in their knowledge scores. In line with these results, Zhong et al. [44] in China and Pal et al. [24] in India did not show a significant difference in the knowledge score according to gender. However, a study in Egypt showed the level of knowledge of women to be significantly higher than that of men [48]. Moreover, in Ebrahimi et al. in Mashhad [40], Rahmanian et al. in Jahrom [49] showed the level of knowledge of women regarding corona to be higher than that of men.

The patients with a history of diabetes in first degree relatives had more knowledge about COVID-19 than the others. The possible reason for this is that they have been able to get good information about various aspects of diabetes and the threatening conditions of diabetic patients through family members. Consistent with these, Joshi revealed that people with a family history of diabetes had more knowledge about various aspects of diabetes than others [50]. Van der Merwe et al. results showed that people who know their family history of hereditary diseases had more knowledge about these diseases than others and were more successful in diagnosing the disease [51].

Our results revealed that the mean score of the participants based on the type of drug used except in “Knowledge about the transmission of the COVID-19” subscale had a significant difference. In other words, the patients taking metformin had more knowledge about COVID-19 than others. This could be related to the characteristics of the samples, as in the treatment regimen of the majority of patients in our sample, either metformin alone or metformin together with other drugs were prescribed. However, in the results of the effect size index, this relationship has been confirmed in the weak and unstable range. Hence, it is suggested that more studies with a larger number of samples or meta-analysis should be explored to confirm the stability of this finding.

Our findings indicate the general attitude of patients towards the disease in the optimal level. However, 8.7% of the samples were against the fact that it is possible for them to catch corona (22.7% did not have an opinion). These findings indicate that not all society is aware of the risk of the disease and do not consider themselves exposed to it. Hence, if people do not accept that they are susceptible to the disease and may be harmed by it, they are less likely to take preventive actions. The results of another study in Iran indicated that only 60% of people considered themselves to be at risk of contracting corona, whereas almost all adults are at risk of contracting corona according to the literature [41]. In a study on T1DM, about 91.3% of the participants believed that they are at higher risk of contracting COVID-19 [42]. Inconsistent with our study, Nasirzadeh and Aligol study on the people of Qom showed that the perceived sensitivity among the people about COVID-19 was at a high level [52]. In our study, 55.5% of the participants held the belief that using herbal and traditional medicines is more effective in the treatment of corona than medical treatments, and 21.6% believed that the use of opium can prevent corona. Moreover, 38.4% chose yes in response to the item “Health and illness are in the hands of God and we cannot do much to prevent the corona virus.” Having misconceptions about corona and other diseases is not just specific to this study, and a review of studies shows that some misconceptions about this disease continue [53]. Hence, in Kakemam, 47% of people believed that the virus is destroyed by salt solution, and 58% believed that the virus is transmitted through wild animals [41]. All these cases show the poor attitude of the society about COVID-19. Any misconceptions on the disease of COVID-19 could result in a sharp increase in the incidence of the disease. Hence, it is necessary to provide more comprehensive and detailed training through the media, doctors, health workers, researchers and other stakeholders to affect the society's attitude towards COVID-19.

According to the results, the mean practice score of patients with T2D in the field of preventive behaviors against COVID-19 was reported as optimal. However, a more detailed analysis of the results in the practice section showed 47% in response to “washing hands with soap and water for 20 s,” 51% in response to “regular use of a mask,” 57.7% in response to “complying with quarantine and staying at home,” 59.4% in response to “avoiding being in crowded places and using a double-layer mask in these places,” and 56% in response to “following their usual diet recommendations” chose the option “I do to some extent” that shows the diabetic patients participating in this study do not pay serious attention to the prevention and care protocols of COVID-19. In explaining the poor practice of patients in some items such as “failing to comply with quarantine and staying at home”, one of the possible reasons can be affected by being forced to do daily tasks and social and economic issues. In some cases, presence in crowded places has become unavoidable for some sections of the society, however, in cases such as “non-regular use of masks”, other reasons such as the weak implementation of restrictive laws by the government during the pandemic have been effective.

The results indicated that the mean practice score of urban participants was significantly higher than that of rural participants, and in terms of the effect size index, the study showed a very strong relationship, but it did not have the desired stability. Moreover, according to multiple regression analysis, residence and knowledge about virus diagnosis and treatment were the most important predictors of patients' practice scores in the field of preventive behaviors against COVID-19. Thus, more detailed planning should be done according to residence to determine the educational needs of patients with diabetes and other sections of the society.

According to the results, patient practice regarding COVID-19 did not have a significant relationship with their knowledge of covid-19. However, it had a positive and significant relationship with their attitude. As already stated, a part of people's practice can be affected by determinism because of the needs of fasting and sometimes due to the lack of restrictive laws. Nonetheless, it indicates that merely improving their knowledge is not enough to improve the practice of patients.

## Conclusion

The good level of knowledge and attitude and practice of people towards COVID-19 were good in our study. However, in some cases there were answers showing poor knowledge (considering smoking and drug use to be effective in eliminating the coronavirus, transmission of the virus through insulin injection needles and so on), false beliefs and attitudes (such as the better effect of herbal and traditional medicines in the treatment of corona than medical treatments, preventing opium from contracting corona, our inability to prevent the Coronavirus disease) and average status of practice regarding various aspects of the COVID-19. Residence was a strong predictor of T2DM practice in terms of protective behaviors against COVID-19. Thus, educational needs evaluation according to the place of residence is recommended, especially for T2DM patients who live in rural areas.

### Strength and limitation of the study

This is the first study carried out among Iranian T2DM patients living in the southern regions of the country regarding KAP towards COVID-19, and an acceptable sample size was collected from both rural and urban populations. This could be seen as one of the strengths of the present study.

Among the limitations of the study was that the possibility of recall bias could not be eliminated because of the nature of the self-reporting questionnaire. Moreover, because of the wide range of information about COVID-19, it was impossible to evaluate all of them comprehensively in the three dimensions of KAP.

### Implications of study results

- Residence was a strong predictor of type 2 diabetes mellitus patients’ practice in terms of protective behaviors against Covid-19. Hence, educational needs evaluation based on residence is recommended, especially in rural T2DM patients.
- Although two years have passed since the beginning of the epidemic of covid-19, our results showed that hesitancy towards the various aspects of the Covid-19 disease (Ways of transmission, Care and Prevention, and treatment of the disease).
- The results show that not all the society is aware of the risk of the Covid-19 disease and they do not consider themselves exposed to it; therefore, they are less likely to engage in preventive behaviors. This confirms the importance of designing educational interventions more than in the past.

## Data Availability

The datasets used and/or analyzed during the current study are available from the corresponding author upon reasonable request.

## Abbreviations

GK: General knowledge about COVID-19
KNCVD: Knowledge about the nature of the coronavirus and the disease
KT: Knowledge about the transmission of the coronavirus
KCP: Knowledge about the care and prevention of the coronavirus
KDT: Knowledge about coronavirus diagnosis and treatment
GA: General attitude towards Covid-19
AC: Attitude towards CONTRACTING the disease
ASMD: Attitude towards severity and mortality of the disease
ADP: Attitudes towards disease prevention
ADT: Attitudes towards disease treatment
PC: Practice regarding COVID-19
T2D: Type 2 diabetes
